# Evaluation of the Protein Profile of a *Saccharomyces cerevisiae* Strain Immobilized in Biocapsules for Use in Fermented Foods

**DOI:** 10.3390/foods13233871

**Published:** 2024-11-29

**Authors:** Juan C. García-García, Miguel E. G-García, Juan C. Mauricio, Juan Moreno, Teresa García-Martínez

**Affiliations:** 1 Department of Agricultural Chemistry, Edaphology and Microbiology, Agrifood Campus of International Excellence ceiA3, University of Córdoba, 14014 Córdoba, Spain; p22gagaj@uco.es (J.C.G.-G.); qe1movij@uco.es (J.M.); mi2gamam@uco.es (T.G.-M.); 2 Department of Cell Biology, Physiology and Immunology, University of Córdoba, 14014 Córdoba, Spain; b62gagam@uco.es

**Keywords:** free yeasts, immobilized yeasts, filamentous fungus, two-dimensional gel electrophoresis, proteins

## Abstract

Yeast biocapsules are a novel immobilization technology that could be used in fermentation processes. They are spherical structures consisting of yeast cells encapsulated and attached to the hyphae of a filamentous fungus. Yeast biocapsules offer a cutting-edge approach to cell immobilization, with significant potential for advancing fermented food production. By enhancing fermentation control, improving product quality, and increasing process efficiency, these biocapsules represent a key innovation in food fermentation technology, particularly in the production of alcoholic beverages such as beer and wine. Proteomic analysis of two-dimensional gels was carried out to study changes in proteins expressed in (i) co-immobilized yeast cells, and (ii) free-format yeast cells. This analysis showed that the proteins expressed in co-immobilized yeast cells played critical roles in DNA repair, cell cycle regulation, protein synthesis, and translation, whereas the proteins expressed by free yeast cells were mainly related to glycolysis. These findings suggest a defense response of the co-immobilized yeast against fungal interactions, involving regulatory mechanisms at the DNA, RNA, and protein levels. This study opens new avenues for exploring yeast–fungus co-immobilization, including stress responses, the nature of the binding polymers, and the proteomics of biocapsules. Additionally, investigating natural co-immobilization mechanisms between various microorganisms could uncover further biotechnological applications and biocatalytic activities.

## 1. Introduction

Yeast immobilization has become an important technique in fermentation processes, offering numerous advantages to produce fermented foods, such as beer and biofuels. Beer provides soluble fiber, minerals, and vitamins as well as showing beneficial health effects [1,2,3]. Among immobilization methods, yeast biocapsules represent a novel and promising approach. The original biocapsules were formed by the co-immobilization of a flor yeast (*Saccharomyces cerevisiae*) and a filamentous fungus (*Penicillium chrysogenum*), resulting in hollow spheres with yeast cells both inside and attached to the fungal hyphae [4,5,6]. The use of flor yeasts, traditionally associated with the production of Sherry-type wines, has expanded to various applications, including the biological aging of red wines, the production of sparkling wines, and the formation of yeast biocapsules [4,7,8]. Proteomic analysis provides a powerful tool to investigate the complex interactions and adaptations that occur within yeast biocapsules. The study of proteomic profiles in immobilized yeasts is of significant interest for both scientific research and industrial applications. This approach offers valuable insights into yeast behavior and adaptation under various conditions, particularly in comparison to free yeast cells. By providing a comprehensive view of protein expression, proteomics can provide insights into the co-immobilization process, metabolic capabilities, stress responses, and potential applications of immobilized yeast cells. Although several proteomic techniques are available, two-dimensional gel electrophoresis (2-DE) remains a valuable method for comparative proteomic studies [9,10,11]. It allows the visualization of thousands of intact proteins while preserving information on their abundance, isoforms, and post-translational modifications (PTMs).

## 2. Materials and Methods

### 2.1. Microorganism Strains and Yeast Biocapsule Formation Conditions

*Penicillium chrysogenum* H3 (UCDFST 22-448) and *Saccharomyces cerevisiae* G1 (ATCC: MYA-2451) were used in this study, both of which were isolated by the Microbiology Department of the University of Córdoba (Spain). The *P. chrysogenum* strain was isolated from the laboratory environment, while the *S. cerevisiae* strain was obtained from a flor velum (Montilla-Moriles, Córdoba, Spain).

Before forming yeast biocapsules, *S. cerevisiae* cells were first streaked on YPD agar (1% yeast extract, 2% peptone, 2% glucose, and 2% agar) and then cultured overnight in YPD liquid medium at 28 °C with shaking at 175 rpm. *P. chrysogenum* spores were cultivated on a sporulation medium containing 1.7% (*w*/*v*) corn meal agar (BD, Difco, Sparks Glencoe, MD, USA), 0.1% yeast extract, 0.2% glucose, and 2% agar.

**Figure 1:**
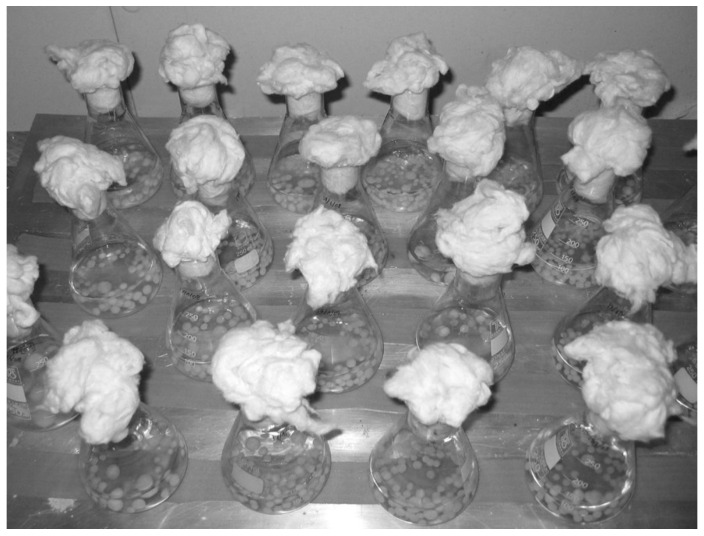
Formation of yeast biocapsules 7 days after inoculation of microorganisms in the formation medium on an orbital shaker.

Biocapsules were produced in a formation medium using Yeast Nitrogen Base (YNB) without amino acids (BD, Difco, Sparks Glencoe, MD, USA), supplemented with 5 g/L gluconic acid as a carbon source, and buffered to pH 7 with Na_2_HPO_4_ and KH_2_PO_4_. The medium was inoculated with both yeast cells (4 × 10^6^ yeast cells/mL) and fungal spores (3 × 10^6^ spores/mL) in 250 mL Erlenmeyer flasks. Incubation was carried out at 28 °C with shaking at 150 rpm for 7 days, resulting in spontaneous co-immobilization and formation of yeast biocapsules without external support, as shown in Figure 1 [4]. Free yeast cells were inoculated in the same medium as the biocapsules and then treated in the same way in parallel.

### 2.2. Cell Preparation

The co-immobilized yeast cells were removed from the biocapsules, first by manual mechanical disaggregation of the hyphae using a mortar and pestle, but only gently to separate the hyphae, and then by vigorous shaking on a shaker (Heidolph DSG 304, Techtrader, Artarmon, NSW, Australia) in the presence of 100 mM NaCl solution for 30 min at 4 °C. The yeast cells were then separated from the filamentous fungus hyphae by two successive filtrations with Millipore filters (Burlington, MA, USA) of different pore sizes, the first with a 180 μm diameter and the second with a 30 μm diameter. Yeast cells were harvested by centrifugation at 7000 rpm for 10 min at 4 °C in a Beckman Coulter J2-HS centrifuge (Brea, CA, USA) with a JA-14 rotor. Free yeast cells were treated in the same way and collected by centrifugation under the same conditions as above.

### 2.3. Protein Extraction

Yeast cell extracts were prepared through cell lysis and protein solubilization. The process began with centrifuging suspended cells and discarding the supernatant. The resulting pellet was washed twice with bidistilled water. Each sample was then suspended in sterile water containing dithiothreitol (DTT, Merck, Darmstadt, Germany) and crushed with glass beads (Sigma-Aldrich, San Luis, MO, USA).

The sample underwent denaturation using a buffer containing sodium dodecyl sulfate (SDS, Panreac, Barcelona, Spain) in Tris-HCl. A second buffer containing thiourea, urea, CHAPS (Sigma-Aldrich, San Luis, MO, USA), DTT, Biolytes 3–10 (Bio-Rad, Hercules, CA, USA), and protease inhibitors (Roche, Paris, France) was then added. The mixture was shaken at room temperature for 2 h, centrifuged, and the supernatant was collected for storage or further purification.

Protein extracts were purified using trichloroacetic acid in cold acetone containing DTT. After overnight precipitation at −20 °C, the protein pellet was collected by centrifugation, washed with cold acetone containing DTT, and finally dissolved in the second buffer. The protein concentration in each cell extract was determined using the Bradford method with the Bio-Rad RC-DC protein assay (Bio-Rad, Hercules, CA, USA).

The prepared samples were collected in liquid nitrogen and stored at −80 °C until they were ready for isoelectric focusing (IEF). This method ensured the preservation of protein integrity and allowed for subsequent proteomic analysis. For more details see Maestre et al. [12].

### 2.4. Two-Dimensional SDS-PAGE

The first dimension of protein separation was carried out using IPG strips from Bio-Rad, measuring 17 cm in length with a linear pH gradient of 3–10. Each strip was rehydrated with 150 µg of protein sample dissolved in 300 µL of buffer 2 [13,14]. Isoelectric focusing (IEF) was performed using a Bio-Rad Protean IEF cell. The focusing process was conducted at a constant current of 50 µA per strip, continuing until a total of 40,000 volt-hours (V × h) was reached. The specific program used for IEF is detailed in Table 1.

For the second dimension SDS-polyacrylamide gel electrophoresis (SDS-PAGE), the strips underwent a two-step equilibration process [15]. First, they were soaked for 10 min in equilibration buffer a, containing Tris-HCl, urea, SDS, glycerol, and DTT. This was followed by another 10 min soak in buffer b, which had a similar composition but with iodoacetamide replacing DTT. The SDS-PAGE was then performed using 13% polyacrylamide gels in a Protean Plus Dodeca Cell (Bio-Rad, Hercules, CA, USA). A constant current of 60 mA per gel was applied. For more details see Maestre et al. [12].

### 2.5. Gel Staining and Image Acquisition

Preparative gels were stained with Coomassie Brilliant Blue G-250 (Merck, Darmstadt, Germany). Images were captured using a GS-800 calibrated densitometer and analyzed with PDQuest 8.0.1 software. The guided protein spot detection method was employed, using a minimum criterion of 10-fold over background for presence/absence of spots.

Normalized spot volumes were calculated for each spot by dividing individual spot intensity by a normalization factor based on the total quantity in valid spots for each gel. These values were used to identify significantly differentially expressed spots, defined as those with at least a two-fold increase or decrease. Statistical significance was determined using ANOVA with a homogeneity of variance test, considering spots with a *p*-value < 0.05 and a fold change (FC) > 2 as significant.

To enhance the reliability of the matching, automated analysis was followed by spot-by-spot visual validation. Experimental isoelectric points (pI) were determined using a 3–10 linear scale across the entire length of the IPG strip. Molecular weight (Mw) values were calculated by comparing mobility with protein standard markers that run in a separate lane on the gel. For each spot, the average protein amount, standard deviation (SD), and coefficient of variation (CV) were calculated.

### 2.6. In-Gel Digestion of Proteins and Sample Preparation for MS Analysis

The protein identification process was carried out at the Central Research Support Service, Proteomics area of the University of Córdoba (SCAI).

Protein spots were excised using an Investigator™ ProPic station (Genomic Solutions, Ann Arbor, MI, USA). Then, the excised spots were subjected to enzymatic digestion with trypsin using an automated protein digestion station, Investigator™ Progest apparatus (Genomic Solution). The peptides resulting from the trypsin digestion of the proteins of interest are purified using a C18 resin microcolumn (ZipTip, Millipore), eluting directly with a matrix solution (3 mg/mL alpha-cyano-4-hydroxycinnamic acid in 70% acetonitrile/0.1% TFA) on the MALDI plate in a volume of 1 µL [16].

### 2.7. MALDI PMF, TOF/TOF Acquisition, and Database Searching

After crystallization on the plate, the samples were analyzed by MALDI-TOF/TOF mass spectrometry (MS) to obtain the peptide fingerprint in a spectrometer of masses (4800 Plus MALDI TOF/TOF Analyzer (AB Sciex, Madrid, Spain)) equipped with delayed extraction, reflector and in positive mode, in a mass/charge (*m*/*z*) range of 800 to 4000 Da, with an acceleration voltage of 20 kV. Internal calibration of the spectra was performed using the mass/charge ratios (*m*/*z*) of the peptides resulting from the autolysis of porcine trypsin (M + H^+^ = 842.509, M + H^+^ = 2211.104), thus obtaining precision in the *m*/*z* measurement of ±20 ppm. For each sample, fragmentation spectra (MS/MS) of the eight most intense *m*/*z* were obtained. Protein identification was performed by combining MS spectra and their corresponding MS/MS on databases public protein sequences (NCBInr, Uniprot), using MASCOT v2.0 (MatrixScience Ltd., London, UK; http://www.matrixscience.com (accessed on 6 March 2023) as a search engine, integrated into the GPS ExplorerTM v3.5 program (AB Sciex). Protein spots were identified in the Swiss-Prot database and the TrEMBL database for the yeast proteome of *S. cerevisiae* (www.expasy.ch/sprot (accessed on 6 March 2023)) [11].

### 2.8. Microscopy

Yeast biocapsules were analyzed using both scanning electron microscopy (SEM) and transmission electron microscopy (TEM) techniques.

For SEM analysis, the walls of medium-sized biocapsules underwent dehydration using an ethanol series. The samples were then coated with gold using a Bal-Tec SCD005 sputter coater. Observation and imaging were performed using a Jeol 6300 scanning electron microscope.

For TEM sample preparation, fresh wall biocapsule samples were fixed for 6–8 h in a mixture of 2% paraformaldehyde and 2.5% glutaraldehyde in 0.1 M cacodylate buffer (pH 6.8). After washing with the same buffer, samples were post-fixed in 1% aqueous osmium tetroxide for 1 h. They were then washed again and dehydrated using a graded ethanol series before being embedded in LR White resin. The entire process was conducted at 4 °C following standard protocols, and the LR White resin was polymerized at 60 °C. Thin sections were prepared and mounted on nickel grids, followed by staining with 4% aqueous uranyl acetate and Reynold’s lead citrate. Finally, the prepared samples were viewed and photographed using a Philips EM 300 transmission electron microscope.

### 2.9. Statistical Analysis

The protein spots obtained in at least 50% of some of the samples were represented in a Veen diagram. A one-way analysis of variance (ANOVA) was performed on the protein spots that were found in 100% of all samples, along with their quantification values, applying the homogeneity of variance test, with a *p*-value < 0.05 and a fold change (FC) > 2. The cut-off value was set at −log(*p*-value) = 1.3, being *p*-value = 0.05. The criterion was to discriminate between significant and non-significant values. Statistical analyses were studied using Prism 9.0 software (GraphPad Software, La Jolla, CA, USA). Three replicates were performed.

A study was performed by constructing protein–protein interaction network maps (INMs) using the STRING v12.0 database, using the term “Biological processes” from the Gene Ontology database. High and medium confidence interactions were used (score = 0.4–0.7), with an FDR < 0.05 as significance value.

## 3. Results

The main purpose of this study is to elucidate the proteomic profile of yeast cells immobilized in biocapsules compared to free yeast cells under identical conditions, using 2-DE coupled to mass spectrometry to identify and characterize proteins involved in the immobilization process and subsequent cellular adaptations, while using string analysis to uncover protein–protein interactions, functional associations, and roles in biological pathways and systems during the co-immobilization process, in an attempt to improve this immobilization system for potential use in the brewing and winemaking process.

### 3.1. Comparative Two-Dimensional Gel Electrophoresis of Yeast Proteins in Free and Immobilized Cells

**Figure 2:**
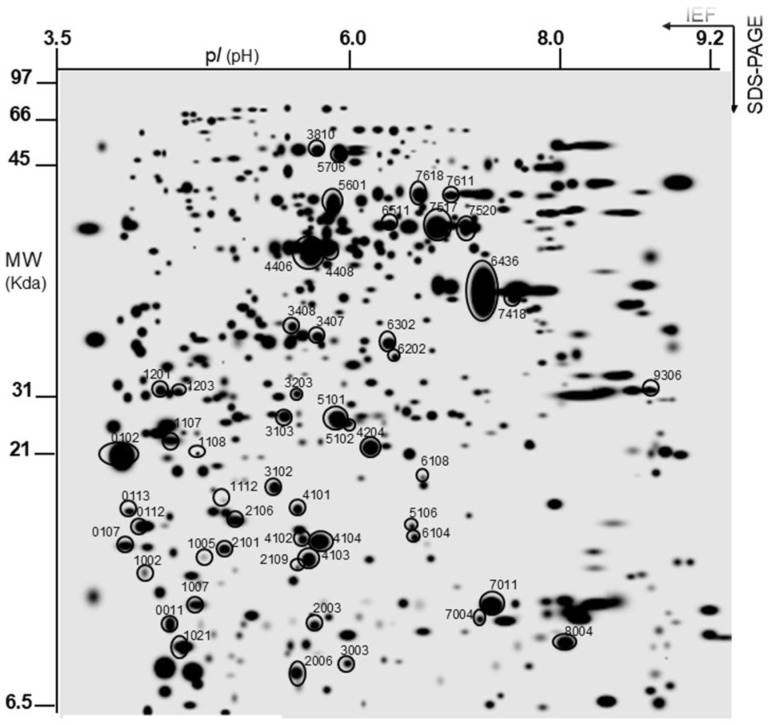
Master gel obtained from the PDQuest-based comparison of 2D electrophoresis gels of co-immobilized and free cells as control. The virtual image of the gel represents all spots as detected in all tested samples. Molecular mass standards are indicated on the left, while the pH range is at the top. Equivalent protein contents (500 µg of protein total) were loaded in each sample. Spots analyzed by MALDI/TOF MS are circled and numbered.

Once the yeast protein extracts were obtained in both conditions, two-dimensional electrophoresis (SDS-PAGE) was performed as indicated in the Materials and Methods section. Subsequently, the gels were analyzed with the PDQuest program to identify the proteins of interest by MS, which were then studied to obtain a general classification of the resulting proteins under the tested conditions. The 2D-PAGE analysis revealed 524 total protein spots characterized in terms of pI and molecular mass (MW, kDa) under the tested conditions. The ‘master gel’ is a virtual gel containing all the individually detected protein spots belonging to all the gels of the same experiment under the studied conditions (Figure 2). The free yeast cell gels were chosen as the reference gel against which the other gels were compared. The circled protein spots are those that showed differential expression, both qualitatively and quantitatively, after comparison and analysis of the spots using the PDQuest 2-D analysis software, version 8.0.1, in both conditions. The proteins considered to be different between the two experimental groups met the following criteria: spots considered for each treatment appeared in at least two of the three replicates for each condition. The results shown are expressed as qualitative and/or quantitative differences in protein expression. For qualitative differences, a qualitative analysis was performed when spots were present in one set of gels from one treatment and absent in the other set of gels from the other treatment, and/or vice versa. For quantitative differences, a statistically significant quantitative analysis was performed, referring to the amount of expressed protein present in the examined conditions.

After a comparative analysis of the gel images with the software, a total of 386 ± 9 spots were analyzed from the immobilized yeast and 483 ± 4 spots from the free-form yeast, and a total of 524 spots between the two conditions, with, therefore, only 53 showing differences in expression, corresponding to both qualitative and quantitative differences between the two studied conditions. These were selected for protein profiling analysis and finally identified as shown in Table 2.

**Figure 3:**
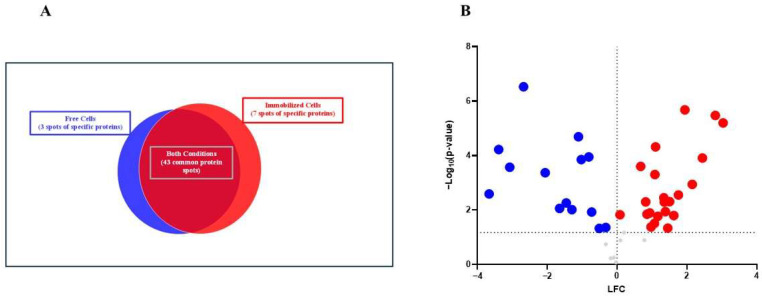
(**A**) Venn diagram representing the number of protein spots of strain G1 in different conditions: Free Cells (blue), Immobilized Cells (red), and common to both (free and immobilized cells; dark red). (**B**) Volcano plot of the log_2_ fold-change of protein spots in immobilized cells vs. protein spots from free cells with significant differences with a *p*-value < 0.05. Proteins upregulated in immobilized cells correspond to red balls (LFC > 0) and downregulated to blue balls (LFC < 0). Proteins without significant differences are represented in grey (*p*-value > 0.05).

Of the 53 spots obtained, 43 were common, 3 were specific to free yeast and 7 were specific to immobilized yeast, as shown in Figure 3A. From the protein spots, the accession numbers of the corresponding proteins were obtained, but in a few cases, the same accession number coincided for different spots, due to two PTM types being found: oxidation (M) and carbamidomethyl (C). The first, the oxidation of methionine, occurs when the amino acid methionine reacts with reactive oxygen species (ROS) from normal metabolism or environmental stress, resulting in the formation of methionine sulfoxide. It is often reversible and can be repaired by cellular mechanisms. The second modification, carbamidomethyl (C), is applied to cysteine residues in proteins during sample preparation for mass spectrometry-based proteomics [17].

There were 43 protein spots in common between the two study conditions. These were subjected to a one-way analysis of variance (ANOVA) using the homogeneity of variance test, with a *p*-value < 0.05 and a fold change (FC) > 2. A total of 36 protein spots with significant differences were obtained and are shown in Figure 3B and Table 3.

Among those that showed quantification peaks in free yeast, some stress-related proteins could be highlighted: PST2, induced by oxidative stress, is located in mitochondria, and SOD1, which detoxifies superoxide, which enters into the nucleus under oxidative stress to promote the transcription of stress response genes, as well as the protein MDH1, located in mitochondria, which is involved in the tricarboxylic acid (TCA) cycle. In addition, proteins with quantification peaks are observed in co-immobilized yeast, of which several stress-related proteins could be highlighted: GRE1, involved in the stress-induced dehydration-rehydration process, AHP1, which reduces hydroperoxides to protect against oxidative damage, and POP3, required for 5.8S rRNA processing at the A3 site and for 5′ and 3′ processing of pre-tRNA, also localized in the cytosol in response to hypoxia. Several RNA-related proteins are also present: GBP2, involved in quality control for export of bound mRNA from the nucleus; SBP1, involved in the transition of mRNAs from translation to an mRNA complex destined for decapping; CWC2, required for cell growth, controls the cell cycle and is involved in the initial step of pre-mRNA splicing; and GUS1, which catalyzes the binding of glutamate to tRNA in a two-step reaction. Other proteins of note include IMH1, which is involved in vesicular transport between an endosomal compartment and the Golgi apparatus; RPL21a, part of the ribosome, a large ribonucleoprotein complex responsible for protein synthesis in the cell; EFT2, a protein that catalyzes ribosomal translocation during protein synthesis; DST1, which allows RNA polymerase II to read through blockages in elongation by stimulating the excision of nascent transcripts stuck at transcription arrest sites; and SCP160, which is involved in the control of mitotic chromosome transmission. As a conclusion, proteomic analysis of the two-dimensional gels in the two studied conditions indicates that the proteins expressed in the co-immobilized yeast cells have functions related to DNA repair and maturation, cell cycle control, and protein synthesis and translation, functions involved in both cell cycle and transcriptional regulation. Therefore, these results could suggest that there is a defensive response of the co-immobilized yeast cells against the fungus under the tested conditions, characterized by regulation at the DNA, RNA, and protein levels.

### 3.2. Protein–Protein Interaction

Two protein–protein interaction analyses were performed using the STRING v12.0 database, one with proteins that showed significant quantitative differences being higher in free yeast cells, together with yeast-specific proteins, and another with proteins that showed significant differences, being higher in immobilized yeast cells, together with proteins unique to this condition.

**Figure 4:**
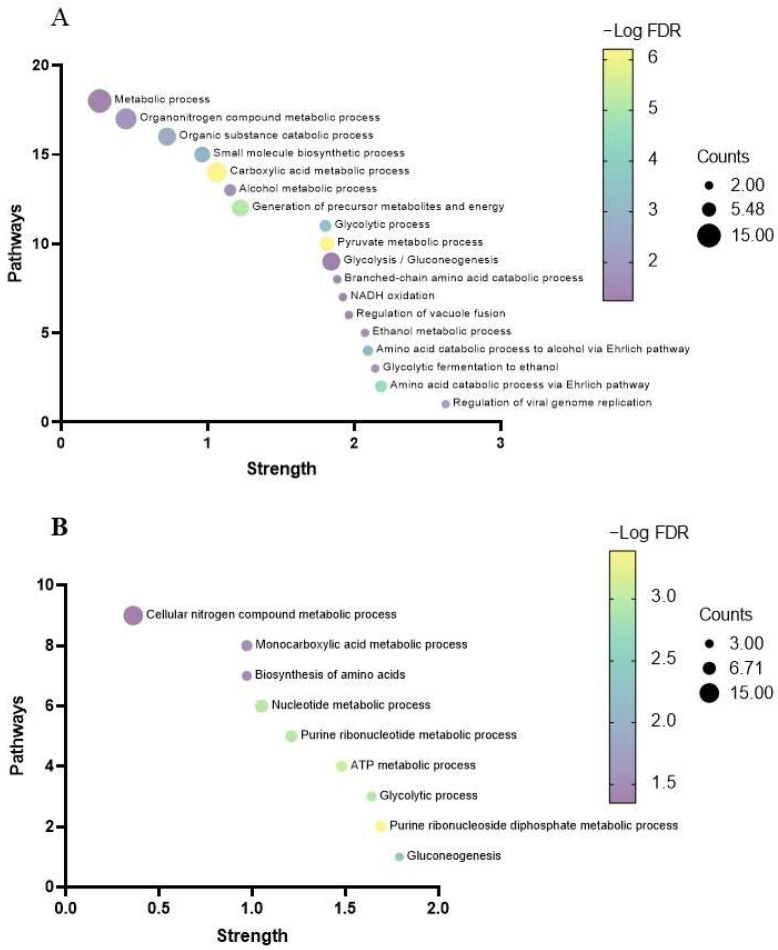
Bubble plot of the enrichment terms of the analysis of the set of proteins most expressed in free cells (**A**) and in immobilized cells (**B**) using the “Biological Processes” term from the Gene Ontology database. The color gradient refers to the significance of each path (FDR < 0.05). The size of the bubbles represents the number of proteins that correspond to each of the pathways.

In Figure 4A, bubble plots show the importance of the biological processes corresponding to the proteins expressed in free cells, in which a greater number of biological processes related to glycolysis can be observed, highlighting glycolytic fermentation to ethanol and amino acid catabolic process via Ehrlich pathway, while in Figure 4B, the processes that stand out are related to genetic material in immobilized yeast cells, specifically, pathways showing high significance were purine ribonucleoside diphosphate, glycolytic, and purine ribonucleotide metabolic processes.

**Figure 5:**
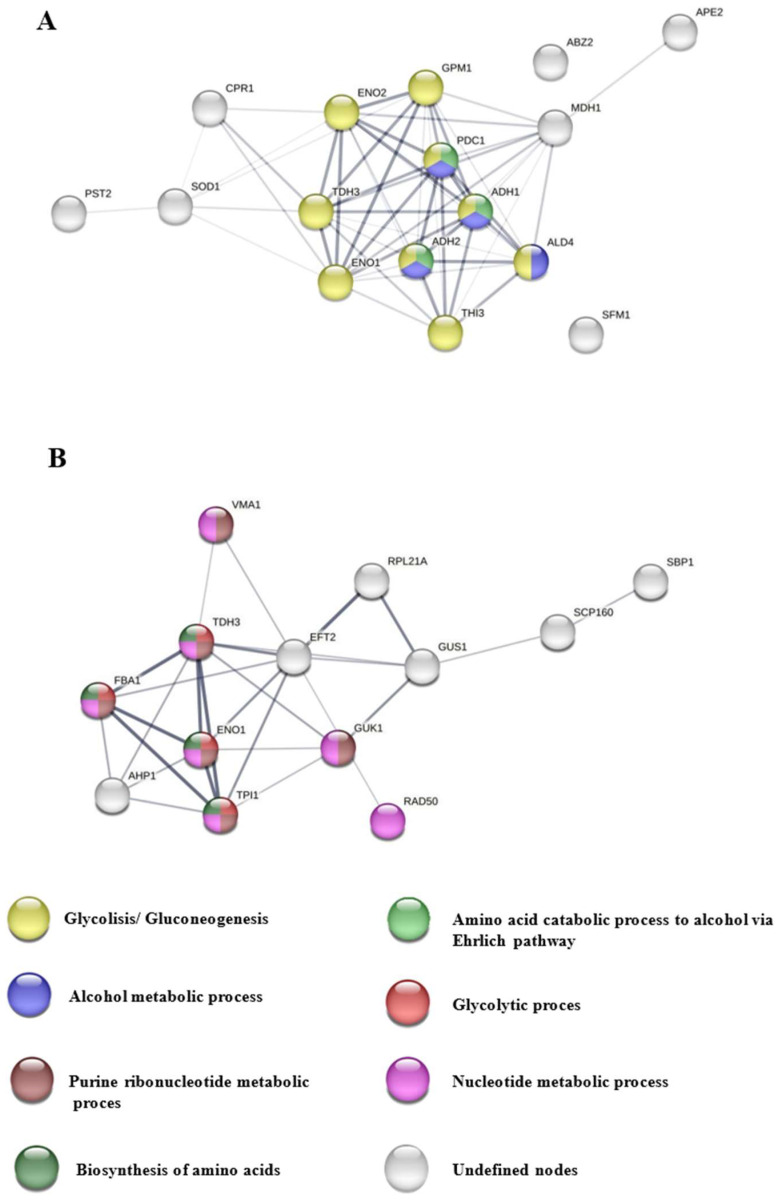
Maps of protein–protein interaction networks (INMs) between the most expressed proteins in free cells (**A**) and in immobilized cells (**B**), built with STRING v12.0, with high and medium confidence, with a significance value FDR < 0.05. The proteins are shown as nodes and the interactions between them are presented by edges whose thickness indicates the strength of each interaction. Nodes of the same color correspond to the same biological process according to the Gene Ontology bases.

Figure 5A shows the interaction network maps (INMs) (55 edges; PPI enrichment *p*-value < 1.0 × 10^−16^) representing a large number of glycolysis/gluconeogenesis-related proteins (yellow nodes), in particular, TDH3, which catalyzes the reaction of glyceraldehyde-3-phosphate to 1,3-bisphosphoglycerate, ENO1 and ENO2, which catalyzes the conversion of 2-phosphoglycerate to phosphoenolpyruvate, GPM1, which is responsible for the conversion of 3-phosphoglycerate to 2-phosphoglycerate, and THI3, which is involved in the catabolism of amino acids, the resulting aldehydes are reduced to alcohols; with the alcohol metabolic process (blue nodes), highlighting ALD4, involved in the formation of acetate during glycolysis; and in the catabolism of amino acids to alcohol via the Ehrlich pathway (green nodes), also belonging to the two biological processes mentioned above, PDC1, involved in the non-oxidative conversion of pyruvate to acetaldehyde and carbon dioxide during alcoholic fermentation, and ADH1 and ADH2, which reduce acetaldehyde to ethanol, the latter being involved in the formation of certain esters.

Figure 5B presents INMs (27 edges; PPI enrichment *p*-value < 0.00325) with proteins related to the purine ribonucleotide metabolic process (purple nodes), including GUK1, which converts GMP to GDP and is required for the growth and elongation of the outer mannose chain of cell wall N-linked glycoproteins, VMA1, involved in the extension of lifespan by methionine restriction in an autophagy-dependent manner; with the nucleotide metabolic process (pink nodes), RAD50, responsible for double-strand break repair, DNA recombination, maintenance of telomere integrity and meiosis, as well as processing of double-strand DNA breaks in vegetative cells; with the glycolytic process (red nodes), and with amino acid biosynthesis (dark green nodes). These proteins are also part of all other biological processes: FBA1 catalyzes the aldolyl condensation of dihydroxyacetone phosphate to form fructose 1, 6-bisphosphate in gluconeogenesis and the reverse reaction in glycolysis. It is also found on the mitochondrial outer surface after oxidative stress. TPI1 is responsible for the conversion of glyceraldehyde-3-phosphate to dihydroxyacetone phosphate and vice versa, whereas TDH3 and ENO1 have already been discussed above.

### 3.3. Macro and Micrographs of a Yeast Biocapsule

**Figure 6:**
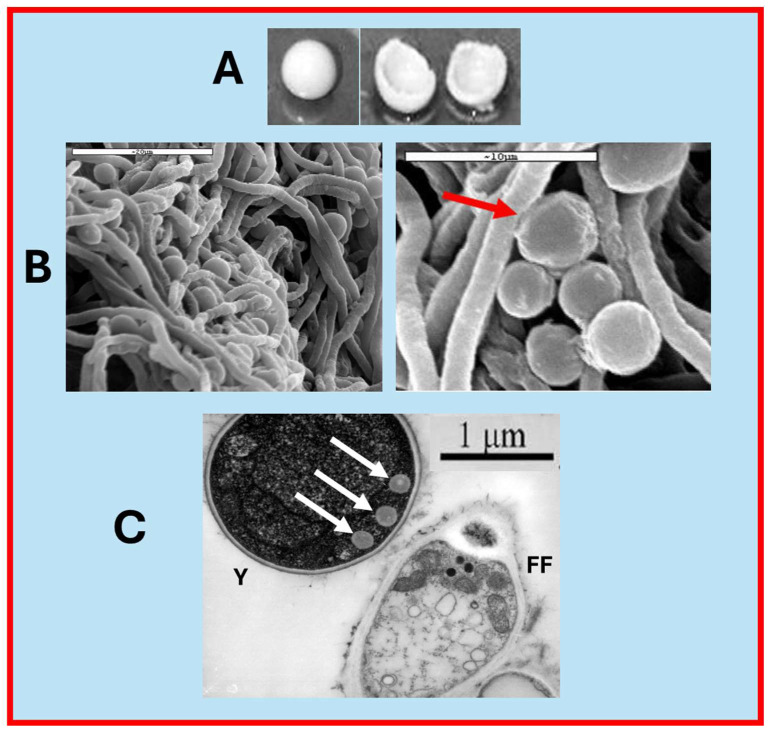
Different images of yeast biocapsules. (**A**) Yeast biocapsules observed with the naked eye, 1.5 cm diameter. (**B**) The close relationship between yeast cells and fungal hyphae can be observed under a scanning electron microscope (SEM), as shown by the red arrow. (**C**) The transmission electron microscope (TEM) image shows the presence of polarized structures towards the fungal hyphae, white arrows. Y: Yeast; FF: Filamentous Fungus.

To the naked eye, a freshly formed yeast biocapsule is spherical and hollow (Figure 6A). Scanning electron microscopy (SEM) shows the tight junctions between the yeast cells and the hyphae of the filamentous fungus (Figure 6B), and transmission microscopy (TEM) shows the presence of polarized structures in the area of contact with the hyphae (Figure 6C).

## 4. Discussion

Yeast biocapsules represent an innovative and sustainable biomaterial that offers a food-safe alternative to conventional immobilization carriers, which are often synthetic or heavily processed. Recent optimization efforts aimed at enhancing cell loading onto the mycelium have yielded significant advancements in both applied and fundamental scientific understanding of inter- and intracellular interactions. The latest studies have extensively documented the complex interplay between fungi and various microorganisms, including microalgae, bacteria, yeasts, and even animal cells [18,19,20,21,22]. This research has not only expanded our knowledge of microbial ecology but also opened up new possibilities for biotechnological applications. Despite this wealth of knowledge, proteomic studies in this area remain limited. Comparative proteomics of free and immobilized cells in yeast biocapsules is crucial for enhancing co-immobilization techniques and understanding symbiotic phenomena between yeasts and filamentous fungi.

Our results align with previous proteomic studies on yeast biofilms (flor yeast) under various conditions. Despite differences in the growth medium—our biocapsule-forming medium lacked fermentable sugars—we observed similar patterns in differential protein expression to those reported by Moreno-Garcia et al. [23]. Key proteins related to stress responses, metabolism, and cellular respiration were identified in both studies. The identification of proteins such as MDH1 (involved in the TCA cycle) and SOD1 (which detoxifies superoxide) corroborates previous observations of oxidative stress responses in yeast biofilms. Stress-related proteins like GRE1 (associated with dehydration–rehydration stress) and AHP1 (linked to oxidative damage) further support the importance of oxidative metabolism in yeast adaptation to biofilm conditions.

STRING database analysis revealed a strong association with glycolytic processes in free yeast cells, consistent with previous studies highlighting the significance of glyoxylate and TCA cycles in biofilm yeast metabolism. Proteins like ALD4, which is involved in acetate formation, and PDC1, which contributes to pyruvate conversion, underscore the critical role of maintaining energy production through glycolysis and related pathways in both free and co-immobilized yeast.

Free yeast cells grew minimally due to the lack of fermentable sugars, relying on energetic glycolysis of reserve materials. In contrast, co-immobilized cells showed slightly more growth, potentially benefiting from energy and reserves provided by the filamentous fungus [24]. The upregulation of proteins related to genetic material maintenance (such as GBP2) and protein synthesis (like RPL21a) in co-immobilized yeast suggests a shift towards maintaining cellular structure and genetic stability.

Our findings contribute to a broader understanding of yeast biofilm formation and cellular adaptation in different environments. They highlight the critical role of oxidative metabolism and stress adaptation in yeast, particularly under immobilized conditions. Yeast biocapsules have shown promise in various fermentation processes, including wine, beer, wastewater treatment, and ethanol production [25,26,27]. They offer advantages such as reduced energy consumption and wine losses in sparkling wine production; facilitation of yeast cell recovery and reuse for multiple fermentation batches; and altered chemical and sensory profiles in beer production; as well as reduced ethanol consumption in sherry wine production. These findings support the potential use of filamentous fungi as a novel cell immobilization biomaterial for food technology applications.

Future research should focus on quantifying differences in mRNA–protein correlation between immobilized and free yeast, investigating the potential parasitic nature of yeast-fungal interactions in co-immobilization systems, and exploring the broader implications of these findings for biotechnology and the fermentation industry [20,21,28,29].

## 5. Conclusions

This proteomic study shows significant differences in the two profiles (free and immobilized cells) and reveals mechanistic details of the cellular responses based on the expression of proteins involved in metabolic pathways during co-immobilization. The results indicate that co-immobilized yeast cells induce a multifaceted defense response against filamentous fungus. This response involves several protective mechanisms operating at different levels, resulting in a comprehensive shield against the fungal threat. This study contributes to our understanding of the molecular basis of yeast immobilization in biocapsules and provides valuable insights for optimizing their production and application in biotechnological processes such as fermented foods.

Future research should focus on optimizing these co-immobilization systems and exploring the wider biotechnological applications of these findings, particularly in the fermentation and bioprocessing industries.

## Figures and Tables

**Table 1:** Protean^®^ i12^TM^ IEF system condition for IEF.

Step	Voltage	Gradient	Current	Value	Units
1	50	Rapid	50	16:00	HH:MM
2	500	Rapid	50	1:00	HH:MM
3	1000	Rapid	50	1:00	HH:MM
4	8000	Linear	50	00:30	HH:MM
5	8000	Linear	50	7:00	HH:MM
6	500	Rapid	50	10,000	Volt H

**Table 2:** Molecular weights and isoelectric points of the protein spots with differential expression.

Spot	Mw (KDa)	pI	Spot	Mw (KDa)	pI
9306	24.77	8.31	4104	16.88	5.75
8004	15.02	7.25	3407	31.42	5.74
7418	34.91	6.61	4406	39.98	5.70
7011	14.84	6.45	4103	16.28	5.69
6436	35.64	6.43	4102	16.95	5.65
7004	14.45	6.41	3203	23.34	5.62
7520	42.62	6.34	4101	18.01	5.62
1005	68.36	6.33	2109	16.01	5.62
7611	47.83	6.31	3408	32.27	5.59
7517	42.70	6.27	3103	21.90	5.54
6108	18.18	6.22	3102	18.80	5.48
7618	48.96	6.21	2106	17.60	5.24
6104	17.01	6.19	2101	16.32	5.16
5106	17.38	6.18	1112	18.33	5.15
3003	12.32	6.13	1108	20.13	4.97
6202	28.85	6.12	1007	14.87	4.96
6511	43.07	6.11	1021	37.60	4.86
6302	30.76	6.10	1203	24.57	4.83
4204	20.63	6.07	1107	20.66	4.78
2003	42.72	6.03	0011	11.70	4.77
5706	62.77	5.98	1201	24.72	4.70
5102	21.57	5.96	1002	24.73	4.68
2006	12.00	5.91	0112	17.39	4.57
3810	62.77	5.88	0113	17.93	4.42
5101	21.64	5.87	0107	16.74	4.29
5601	17.38	5.84	0102	20.01	4.16
4408	39.80	5.75			

**Table 3:** Statistical table of protein spots with significant differences with a *p*-value < 0.05 and log_2_ fold change. Proteins upregulated in immobilized cells correspond to LFC > 0 and downregulated to LFC < 0.

Spot	Name	*p*-Value	LFC	Spot	Name	*p*-Value	LFC
v6436	TDH3	0.00258206	−3.6633263	v112	AHP1	0.01498553	0.08458394
v102	PST2	6.031 × 10^−5^	−3.3890829	v3103	TPI1	0.00025005	0.6766392
v3810	PDC1	0.0002681	−3.0730092	v3203	LOH1	0.00504968	0.81956999
v5601	ENO2	2.9804 × 10^−7^	−2.6771181	v2109	YOR1	0.01433111	0.85904338
v6511	ADH1	0.00042748	−2.0578258	v1201	ENO1	0.01290147	0.93557252
v6302	ADH2	0.00876568	−1.6440443	v6104	TDH3	0.04205506	0.97080536
v7517	ADH1	0.00551414	−1.4554519	v4102	DST1	0.03106772	1.06974153
v4406	ABZ2	0.00972757	−1.2916398	v6202	IMH1	0.00050011	1.08182386
v9306	GPM1	2.0318 × 10^−5^	−1.1052806	v1203	FBA1	4.8146 × 10^−5^	1.10195513
v4103	SOD1	0.00014059	−1.0226561	v2003	ENO1	0.01704601	1.16724603
v7011	CPR1	0.00011224	−0.8092900	v8004	TDH3	0.00355368	1.33726275
v3102	APE2	0.01188344	−0.7270379	v3407	TDH3	0.00513855	1.35306687
v7618	ENO1	0.04729845	−0.5089918	v4101	FBA1	0.01154187	1.38566364
v7418	MDH1	0.04369653	−0.3241338	v107	POP3	0.04639082	1.45242457
				v2006	CWC2	0.00491247	1.50260732
				v3408	EFT2	0.01600825	1.62395071
				v5106	TDH3	0.00280403	1.75604349
				v1021	SCP160	2.0904 × 10^−6^	1.94069795
				v11	COS111	0.00114309	2.15384489
				v5101	RPL21A	0.00012364	2.4440878
				v7004	TDH3	3.3638 × 10^−6^	2.8114212
				v6108	GUS1	6.3396 × 10^−6^	3.03573079

## Data Availability

The data used to support the conclusions of this study are available on request from T.G.-M., the principal investigator.

## References

[1] Kaczyński P., Iwaniuk P., Hrynko I., Łuniewski S., Łozowicka B. (2024). The Effect of the Multi-Stage Process of Wheat Beer Brewing on the Behavior of Pesticides According to their Physicochemical Properties. Food Control.

[2] Obradović N., Balanč B., Salević-Jelić A., Volić M., Đorđević V., Pešić M., Nedović V. (2024). Physicochemical Characterization of Polysaccharide–Protein Carriers with Immobilized Yeast Cells Obtained Using the Freeze-Drying Technique. Foods.

[3] Liu S., Liu J., Hou J., Chao N., Gai Y., Jiang X. (2017). Three Steps in One Pot: Biosynthesis of 4-Hydroxycinnamyl Alcohols Using Immobilized Whole Cells of Two Genetically Engineered *Escherichia coli* Strains. Microb. Cell Fact..

[4] García-Martínez T., Peinado R.A., Moreno J., García-García I., Mauricio J.C. (2011). Co-Culture of *Penicillium chrysogenum* and *Saccharomyces cerevisiae* Leading to the Immobilization of Yeast. J. Chem. Technol. Biotechnol..

[5] Nyman J., Lacintra M.G., Westman J.O., Berglin M., Lundin M., Lennartsson P.R., Taherzadeh M.J. (2013). Pellet Formation of Zygomycetes and Immobilization of Yeast. New Biotechnol..

[6] Shah S.S.T.H., Naeem I., Naeem A., Bhutta N.K., Noor F. (2024). Immobilization Techniques for Beverage Production Using Yeast Cell Systems: Challenges, Types, and Future Perspectives—A Mini Review. Food Health.

[7] Ruiz-Muñoz M., Cordero-Bueso G., Benítez-Trujillo F., Martínez S., Pérez F., Cantoral J.M. (2020). Rethinking about Flor Yeast Diversity and its Dynamic in the “Criaderas and Soleras” Biological Aging System. Food Microbiol..

[8] Martínez-García R., Roldán-Romero Y., Moreno J., Puig-Pujol A., Mauricio J.C., García-Martínez T. (2020). Use of a Flor Yeast Strain for the Second Fermentation of Sparkling Wines: Effect of Endogenous CO_2_ Over-Pressure on the Volatilome. Food Chem..

[9] Marcus K., Lelong C., Rabilloud T. (2020). What Room for Two-Dimensional Gel-Based Proteomics in a Shotgun Proteomics World?. Proteomes.

[10] Meleady P., Ohlendieck K. (2023). Two-Dimensional Gel Electrophoresis and 2D-DIGE. Difference Gel Electrophoresis. Methods and Protocols.

[11] Arunima S., Das A., Kalita P.J., Patil R.I., Pandey N., Bhattacharjee M., Sharma B.K., Das D., Acharjee S. (2024). Improved Methods for Total and Chloroplast Protein Extraction from *Cajanus* Species for Two-Dimensional Gel Electrophoresis and Mass Spectrometry. PLoS ONE.

[12] Maestre O., García-Martínez T., Peinado R.A., Mauricio J.C. (2008). Effects of ADH2 Overexpression in *Saccharomyces bayanus* during Alcoholic Fermentation. Appl. Environ. Microbiol..

[13] Görg A., Weiss W., Speicher D.W. (2004). Protein Profile Comparisons of Microorganisms, Cells and Tissues using 2D Gels. Proteome Analysis. Interpreting the Genome.

[14] Bond U., Blomberg A., Querol A., Fleet G.H. (2006). Principles and Applications of Genomics and Proteomics in the Analysis of Industrial Yeast Strains. Yeasts in Food and Beverages.

[15] Bulangalire N., Claeyssen C., Douffi S., Agbulut O., Cieniewski-Bernard C. (2024). A Novel 2D-Electrophoresis Method for the Simultaneous Visualization of Phosphorylated and O-GlcNAcylated Proteoforms of a Protein. Electrophoresis.

[16] Fuentes-Almagro C.A., Prieto-Álamo M.J., Pueyo C., Jurado J. (2012). Identification of Proteins Containing Redox-Sensitive Thiols after PRDX1, PRDX3 and GCLC Silencing and/or Glucose Oxidase Treatment in Hepa 1–6 Cells. J. Proteom..

[17] Liang X., Kaya A., Zhang Y., Le D.T., Hua D., Gladyshev V.N. (2012). Characterization of Methionine Oxidation and Methionine Sulfoxide Reduction using Methionine-Rich Cysteine-Free Proteins. BMC Biochem..

[18] Sun Y., Ali A., Zheng Z., Su J., Zhang S., Min Y., Liu Y. (2022). Denitrifying Bacteria Immobilized Magnetic Mycelium Pellets Bioreactor: A New Technology for Efficient Removal of Nitrate at a Low Carbon-to-Nitrogen Ratio. Bioresour. Technol..

[19] Barzee T.J., El-Mashad H.M., Burch A.R., Franz A.K., Zhang R. (2023). Immobilization of Diatom *Phaeodactylum tricornutum* with Filamentous Fungi and its Kinetics. J. Microbiol. Biotechnol..

[20] Ogawa M., Kermani A.S., Huynh M.J., Baar K., Leach J.K., Block D.E. (2024). Edible Mycelium as Proliferation and Differentiation Support for Anchorage-Dependent Animal Cells in Cultivated Meat Production. NPJ Sci. Food..

[21] Ogawa M., Moreno-García J., Barzee T.J. (2024). Filamentous Fungal Pellets as Versatile Platforms for Cell Immobilization: Developments to Date and Future Perspectives. Microb. Cell Fact..

[22] Saeed Z., Cheirsilp B., Maneechote W., Kongjan P., Jariyaboon R. (2024). Optimizing Bioencapsulation of Yeast Cells by *Aspergillus tubingensis* TSIP9 and Applications in Bioethanol Production Through Repeated-Batch Fermentation. Biocatal. Agric. Biotechnol..

[23] Moreno-Garcia J., Mauricio J.C., Moreno J., García-Martínez T. (2017). Differential Proteome Analysis of a Flor Yeast Strain under Biofilm Formation. Int. J. Mol. Sci..

[24] Ogawa M., Garcia-Martinez T., Bisson L., Mauricio J.C., Moreno J., Moreno-Garcia J. (2020). Mapping the Intracellular Metabolome of Yeast Biocapsules—Spherical Structures of Yeast Attached to Fungal Pellets. New Biotechnol..

[25] Liu M., Qin X., Wu X. (2022). Study on the Technology of Brewing Red Raspberry Wine by using New Immobilized Yeast Technology. Sci. Rep..

[26] Lu Y., Liu T., Bai R., Jia Y., Chen W., Zhao J., Liu Y. (2024). Potential Application of Grape Endophytic Fungus (*Alternaria alternata* MG1) as Bio-Adjunct and Immobilization Carrier of *S. cerevisiae* in Improving Aroma Quality, Polyphenol Profiles, Antioxidant Activity and Color Stability of Merlot Red Wine. LWT—Food Sci. Technol..

[27] Wang L., Yu T., Ma F., Vitus T., Bai S., Yang J. (2019). Novel Self-Immobilized Biomass Mixture Based on Mycelium Pellets for Wastewater Treatment: A Review. Water Environ. Res..

[28] Zheng Z., Ali A., Su J., Huang T., Wang Y., Zhang S. (2021). Fungal Pellets Immobilized Bacterial Bioreactor for Efficient Nitrate Removal at Low C/N Wastewater. Bioresour. Technol..

[29] Ogawa M., Moreno García J., Nitin N., Baar K., Block D.E. (2022). Assessing Edible Filamentous Fungal Carriers as Cell Supports for Growth of Yeast and Cultivated Meat. Foods.

